# Supplementary material for: Hybrid Perovskites Depth Profiling with Variable-Size Argon Clusters and Monatomic Ions Beams
Source: Materials (Basel). 2019 Mar 2;12(5):726. doi: 10.3390/ma12050726 (PMC6427474; doi:10.3390/ma12050726)
Supplement: Supplementary file 1 [file materials-12-00726-s001.pdf]

Supporting Information

Hybrid perovskites depth profiling with variable-size argon clusters and monatomic ions beams

Céline Noël [1], Sara Pescetelli [2], Antonio Agresti [2], Alexis Franquet [3], Valentina Spampinato [3], Alexandre Felten [4], Aldo di Carlo [2], Laurent Houssiau [1] and Yan Busby [1,*]

1 Laboratoire Interdisciplinaire de Spectroscopie Electronique, Namur Institute of Structured Matter, University of Namur, 5000 Namur, Belgium; celine.noel@unamur.be (C.N.); laurent.houssiau@unamur.be (L.H.)

2 C.H.O.S.E. - Centre for Hybrid and Organic Solar Energy, Department of Electronic Engineering, University of Rome Tor Vergata, 00133 Rome, Italy; pescetel@uniroma2.it (S.P.); antonio.agresti@uniroma2.it (A.A.); aldo.dicarlo@uniroma2.it (A.C.)

3 IMEC, 3000 Leuven, Belgium; Alexis.Franquet@imec.be (A.F.); Valentina.Spampinato@imec.be (V.S.)

4 SIAM platform, University of Namur, 5000 Namur, Belgium; alexandre.felten@unamur.be (A.F.)

* Correspondence: busbyan@gmail.com

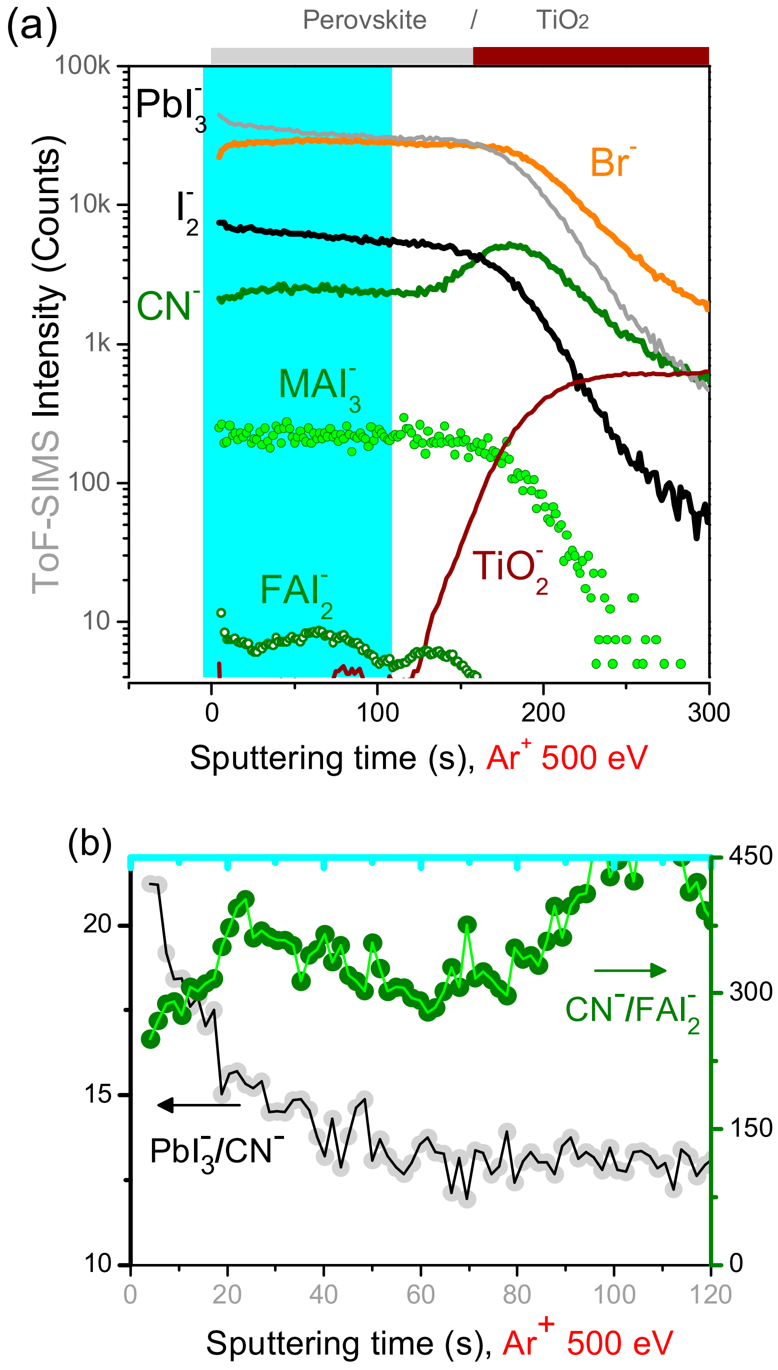

Figure S1. ToF-SIMS depth profiles acquired on Perovskite/cTiO_2 model samples obtained with 500 eV Ar^+ beam. The bottom panel (**b**) refers to the perovskite region and indicate (i) a preferential sputtering of inorganic species (PbI_3^-/CN^- ratio) and (ii) a slowly increasing fragmentation of organic molecules (CN^-/FAI_2^- curve).

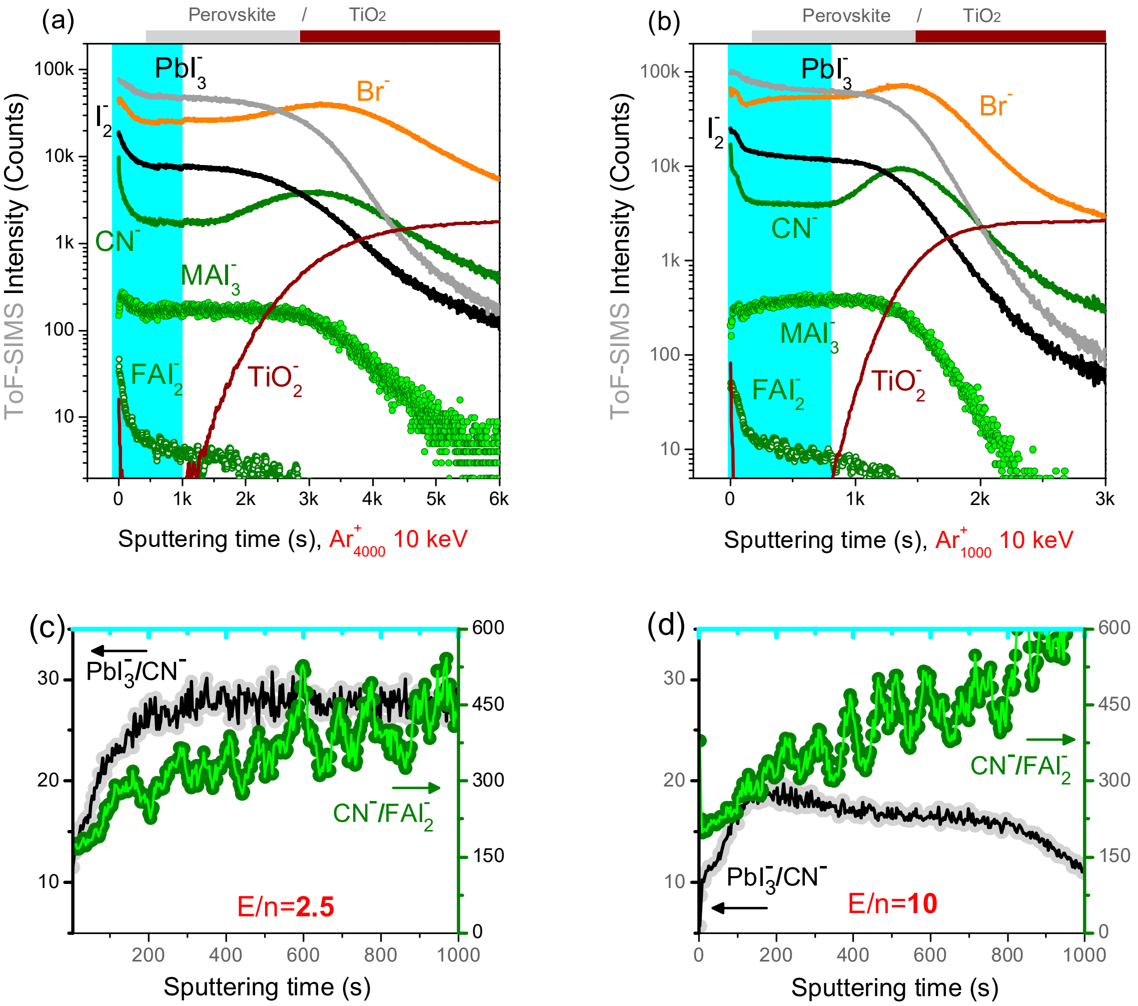

Figure S2. ToF-SIMS depth profiles acquired on Perovskite/cTiO2 model samples obtained with large cluster beams. Constant profiles indicate the non-accumulation of damages. (**a**, **c**) Ar4000+ at 10 keV (E/n=2.5 eV). (**b**, **d**) Ar1000+ at 10 keV (E/n=10 eV). Bottom panels refer to the perovskite region and indicate (i) a higher preferential sputtering of organic species with n=4000 clusters (PbI3-/CN- ratios) and (ii) a similar fragmentation of organic molecules (CN-/FAI2- curves).

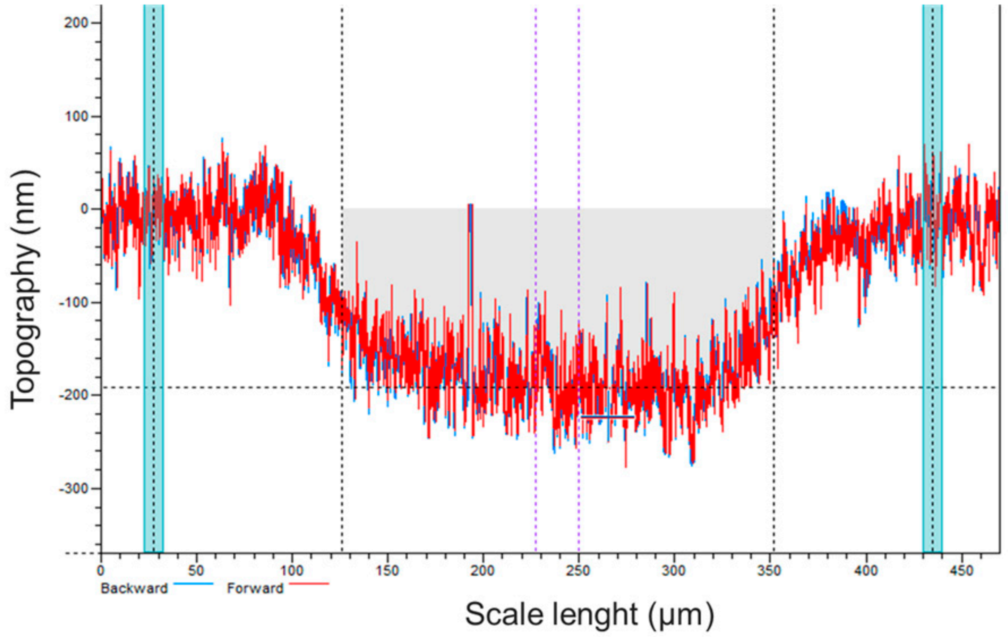

Figure S3. AFM profile of the crater region (grey region) after 40 s of sputtering with Ar_{500}^{+} beam at 20 keV. The crater depth is ~200 nm which corresponds to the sputtering yield of ~5 nm/s.

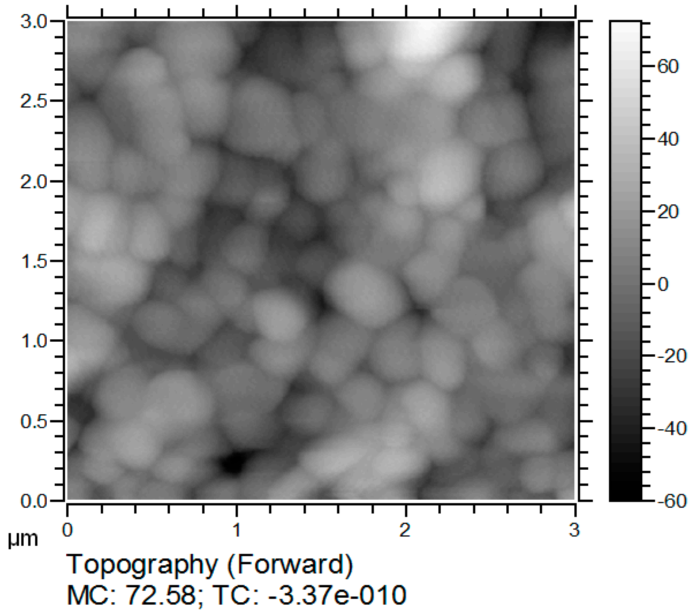

Figure S4. AFM image of the pristine perovskite layer before the sputtering.
